# Exclusive breast feeding in early infancy reduces the risk of inpatient admission for diarrhea and suspected pneumonia in rural Vietnam: a prospective cohort study

**DOI:** 10.1186/s12889-015-2431-9

**Published:** 2015-11-24

**Authors:** Sarah Hanieh, Tran T. Ha, Julie A. Simpson, Tran T. Thuy, Nguyen C. Khuong, Dang D. Thoang, Thach D. Tran, Tran Tuan, Jane Fisher, Beverley-Ann Biggs

**Affiliations:** Department of Medicine, University of Melbourne, Doherty Institute, Parkville, VIC 3050 Australia; Research and Training Centre for Community Development, Hanoi, Vietnam; Centre for Epidemiology and Biostatistics, Melbourne School of Population and Global Health, University of Melbourne, Melbourne, VIC 3010 Australia; Provincial Centre of Preventive Medicine, Hanam Province, Vietnam; The Jean Hailes Research Unit, School of Public Health and Preventive Medicine, Monash University, Melbourne, VIC 3004 Australia; The Victorian Infectious Diseases Service, Royal Melbourne Hospital, Parkville, VIC 3052 Australia

**Keywords:** Diarrhea, Suspected pneumonia, Exclusive breast feeding, Inpatient admission

## Abstract

**Background:**

Acute respiratory infections and diarrhea remain the leading causes of infant morbidity and mortality, with a high burden of both pneunomia and diarrhea in South-East Asia. The aim of the study was to determine antenatal and early infant predictive factors for severe morbidity episodes during the first 6 months of life in Ha Nam province, Vietnam.

**Methods:**

A prospective cohort study of 1049 infants, born to women who had previously participated in a cluster randomized controlled trial of antenatal micronutrient supplementation in rural Vietnam, was undertaken between 28th September 2010 and 8th Jan 2012. Infants were followed until 6 months of age, and the outcome measure was inpatient admission for suspected pneumonia or diarrheal illness during the first 6 months of life. Risk factors were assessed using univariable logistic regression and multiple logistic regression.

**Results:**

Of the 1049 infants seen at 6 months of age, 8.8 % required inpatient admission for suspected pneumonia and 4 % of infants required inpatient admission for diarrheal illness. One third of infants (32.8 %) were exclusively breast fed at 6 weeks of age. Exclusive breast feeding at 6 weeks of age significantly reduced the odds of inpatient admission for suspected pneumomia (Odds Ratio (OR) 0.39, 95 % Confidence Interval (CI) 0.20 to 0.75) and diarrheal illness (OR 0.37, 95 % CI 0.15 to 0.88).

**Conclusions:**

Exclusive breast feeding in early infancy reduces the risk of severe illness from diarrhea and suspected pneumonia. Public health programs to reduce the burden of inpatient admission from diarrheal and respiratory illness in rural Vietnam should address barriers to exclusive breast feeding.

## Background

Globally, pneumonia and diarrheal diseases remain the leading causes of childhood morbidity and mortality and are one of the most common reasons for hospital admission in children in low resource countries [1]. It is estimated that 700 000 child deaths result from diarrheal disease, and 156 million childhood clinical pneumonia cases occur annually, resulting in approximately 2 million deaths in children during the first 5 years of life [1]. The burden of pneumonia and diarrhea is highest in South-East Asia and Africa, with the highest incidence seen in poor and marginalized groups [2]. Recurrent infection and hospitalisation in early life can lead to poor growth and development in childhood, and can impose substantial economic burden on the population and healthcare system. In low income countries, multiple episodes of severe diarrhea in early infancy have been shown to lead to nutritional deficits and long-term growth consequences [3, 4]. The majority of deaths associated with diarrhea and pneumonia occur during the first two years of life, which has important implications for health policy [5, 6].

In Vietnam, the under-five mortality rate is 23.8 per 1000 live births, with much higher rates seen in rural areas [7, 8]. Major causes of death include pneumonia, diarrhea, prematurity and congenital anomalies [7]. The Demographic and Health survey in Vietnam (2002) documented a 20 % prevalence of symptoms compatible with acute respiratory infections (ARI), and an 11 % prevalence of diarrhea in children under three years of age [9]. However, not all cases present to the health system in rural areas and many episodes are not captured by routine data sources; thus the burden of disease may be underreported. Information on maternal and early infant predictors of inpatient admission for these illnesses could greatly assist in the planning of prevention strategies, and early interventions could be targeted towards those most at risk. The primary objective of this study was to identify maternal and early infant factors associated with inpatient admission for suspected pneumonia or diarrheal illness during the first 6 months of life in Ha Nam province, Vietnam.

## Methods

### Study area and population

Ha Nam province is located in North Vietnam, approximately 60 km from Hanoi, in the Red River Delta. Ha Nam has an area of 852.2 km^2^, and a population of approximately 820,100 people with the majority of residents still working in subsistence agriculture [10, 11]. The main ethnic group populating this area is the Kinh, and the province is divided into 112 communes. It is a non-malarious area, with an average rainfall of 1,500 to 2,000 mm and annual average temperature of 23° Celsius. The under 1 year mortality rate in the province is 12.5 per 1000 live births [11]. Health care is provided at the primary health care level through commune health stations (CHS) which are staffed by trained health workers. The team usually consists of a doctor, pharmacist, nurses and a traditional medicine practioner. The CHS provides care and treatment for common diseases and deliveries and carries out health promotion at the village level. Residents are entitled to free services at their local CHS but not at the CHS in other communes. The CHS has a responsibility to the District Health Officer and the Commune People’s Committee, and receives technical guidance from the district hospitals.

### Study design and data collection

A prospective cohort study design was used. The study was carried out in 104 communes and infants eligible for inclusion in the study were those born to women who had previously participated in a cluster randomised trial (Australian New Zealand Clinical Trials Registry: ANZCTR 12610000944033) in the same province. In the original cluster randomised trial, allocation was based on communes, and all communes in the province, other than those in the main provincial town, were randomly assigned to one of three treatment groups. Those in the main provincial town were not included as part of the trial as antenatal services differed from those of the rest of the province, with most deliveries taking place at the provincial hospital. For the original cluster randomised trial, women received either [1] one tablet of iron-folic acid (IFA) taken daily (60 mg elemental iron /0.4 mg folic acid per tablet, 7 tablets per week); or [2] one capsule of IFA taken twice a week (60 mg elemental iron /1.5 mg folic acid per capsule; 2 capsules per week); or [12] one capsule of multiple micronutrients (MMN) taken twice a week (60 mg elemental iron/1.5 mg folic acid per capsule; 2 capsules per week, as well as a variation of the dose of the micronutrients in the UNICEF/WHO/UNU international multiple micronutrient preparation (UNIMMAP) supplement including zinc, iodine, copper, selenium, vitamin A, thiamine, riboflavin, niacin, vitamin B6, vitamin B12, vitamin C, vitamin D and vitamin E [13].

Inclusion criteria for the trial were: residence in trial communes, age >16 y, confirmed pregnancy at <16 wk gestation, and registration with the commune health station. Women were excluded if they had a high-risk pregnancy—multi-fetal pregnancy (confirmed on palpation or ultrasound) or a significant medical condition—or if they had severe anemia (haemoglobin concentration [Hb] <80 g/l) at enrolment. Detailed information on the original cluster randomised trial and inclusion criteria have been reported elsewhere [14]. The trial was approved by the Melbourne Health Human Research Ethics Committee, and the Ha Nam Provincial Human Research Ethics Committee. Written informed consent was obtained from each participant.

Infant morbidity and inpatient admission was assessed at the 6-month post-partum visit to the commune health station, via a structured questionnaire using questions adapted from the World Health Organisation (WHO) Integrated Management of Childhood Illness [15] and modified for use in Vietnam. The primary outcome was inpatient admission for infant diarrheal illness or suspected pneumonia. Interviews were conducted by trained research assistants supervised by experienced Vietnamese and Australian researchers. Information on demographic, and maternal and early infant characteristics, including breast feeding, and maternal and infant nutritional status were collected at enrolment (mean gestational age 12.2 weeks), 32 weeks gestation, 6 weeks and 6 months post-partum. Information on maternal depression was collected at enrolment and 32 weeks gestation. Exclusive breast feeding was defined as- ‘since birth, the child has only received breastmilk (with the exception of oral drops/syrups of vitamins/medicines) and has not been given any other liquids or solids (including water, traditional herbs).’

Maternal mental health was assessed using the Edinburgh Postnatal Depression Scale-Vietnam Validation (EPDS-V). The EPDS-V was translated from English, culturally verified and validated against psychiatrist-administered Structural Clinical Interviews for the Diagnostic and Statistical Manual of Mental Disorders, 4th Edition (DSM IV).

The cut-off point used to detect clinically significant symptoms in this setting was ≥4 [14, 16]. Recurrent depression was defined as a positive EPDS score at enrolment and 32 weeks gestation. Details of the collection of other data used in this study has previously been published [14].

### Case definitions

Suspected pneumonia was defined as any episode of cough or difficulty breathing since birth, and diarrheal infection as any episode of diarrhea (3 episodes of loose stools or bloody stools) since birth [15]. Diarrhea or suspected pneumonia was classified as severe if the child required inpatient admission. All inpatient admissions between birth and 6 months were recorded. Inpatient admission was defined as an admission to any health care facility, that is, commune health centre or hospital during the first 6 months of life for suspected pneumonia or diarrheal illness.

### Statistical methods

Data were analysed using Stata, Version 13 (StataCorp, College Station, TX, USA). Categorical data are presented as percentages with frequency, and continuous data are presented as mean and standard deviation (SD). Univariable logistic regression was initially performed to assess the association between maternal and early infant predictive factors, and inpatient admission for infant morbidity. Multivariable logistic regression models for maternal and early infant factors were then developed including factors with statistically significant univariable associations with the outcome, as well as confounding factors identified *a priori* (maternal age, education, gravidity, infant sex, gestational age, trial intervention, main water source, type of toilet and energy source). To account for clustering at the commune level, robust standard errors were calculated using the Huber-White Sandwich estimator.

## Results

**Fig. 1:**
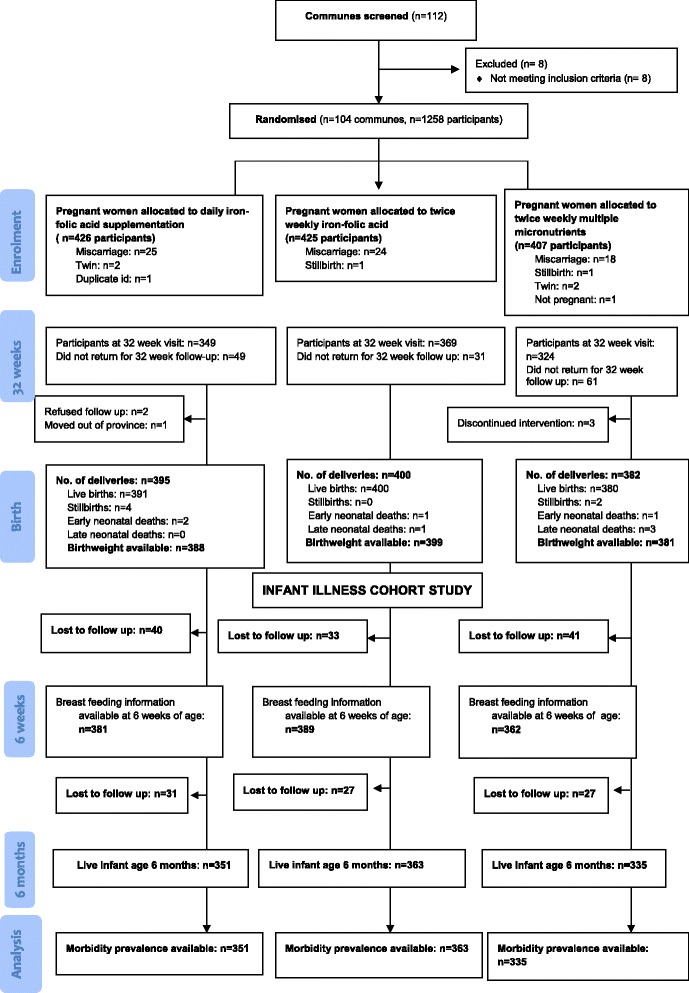
Flow diagram

The 1258 women who were enrolled into the original cluster randomised trial delivered 1177 infants. Six were stillborn resulting in 1171 live born babies. Four early neonatal deaths and four late neonatal deaths occurred, and 114 were lost to follow up in the first 6 months of life. Thus a total of 1049 children were seen at 6 months of age (Fig. 1.)

Demographic and clinical characteristics of the infants are presented in Table 1. The majority of households used rain water (86.5 %), 66.7 % had a flush toilet and gas (79.6 %) was the main source of fuel. At 6 weeks of age, one third of infants (32.8 %) were exclusively breast fed and less than 20 % of infants had ever been exclusively breastfed during the first 6 months.

**Table 1:** Characteristics of mothers at recruitment (baseline measurement) and characteristics of infants at delivery and 6 weeks in Ha Nam province, Vietnam (*n* = 1049)

	Total study population	
Maternal characteristic	Frequency, Mean or Median	(Percentage) [Standard deviation] or {25^th^ – 75^th^ percentile}
Maternal age at enrolment (years)	26.7	[4.9]
<20	41	(3.9)
20–24	340	(32.4)
25–29	426	(40.6)
30–34	161	(15.4)
≥35	81	(7.7)
Maternal height	153.6	[4.7]
<145 cm (short maternal stature)	38	(3.6)
Maternal body mass index	19.9	[1.98]
Underweight (<18.5 kg/m^2^)	272	(26.0)
Normal (18.5-25 kg/m^2^)	761	(72.6)
Overweight (>25 kg/m^2^)	15	(1.4)
Educational level		
Primary school	160	(15.3)
Secondary school	705	(67.2)
University/college	184	(17.5)
Occupation		
Farmer/housewife	561	(53.5)
Factory worker/trader	350	(33.4)
Government official/clerk	138	(13.2)
Main energy source		
Gas	849	(79.9)
Firewood/Straw/Hay	163	(15.4)
Charcoal/coal	30	(2.8)
Electric	20	(1.9)
Type of toilet		
Flush toilet/septic tank	716	(67.4)
Compartment latrine	173	(16.3)
Pit latrine	155	(14.6)
None	18	(1.7)
Main water source		
Rain water	917	(86.4)
Tap/piped water’	87	(8.2)
Well water/river water	56	(5.3)
Gravidity		
Primigravida	328	(31.3)
Multigravida	721	(68.7)
Supplements taken during trial intervention		
Daily IFA supplements	351	(33.5)
Twice weekly IFA supplements	363	(34.6)
MMN supplements	335	(31.9)
Micronutrient status in late pregnancy		
Haemoglobin (g/dL)	12.3	[1.23]
Ferritin (ug/L)	28	{17 to 42}
B12 (pmol/L)	232	{99 to 601}
Folate (nmol/L)	28.7	{6.8 to 90}
Iodine (ug/L)	52.9	{3.3 to 307.4}
Infant characteristics		
Sex		
Male	558	(53.2)
Birthweight (grams)	3157.6	[396.6]
Low birth weight (<2500 gms)	32	(3.1)
Gestational age (weeks)	39.1	[2.0]
Preterm (<37 weeks)	166	(15.8)
Exclusive breast feeding at 6 weeks of age	377	(33.3)
Use of dietary supplements for the child in the first 6 months	117	(11.2)

### Outpatient and hospital admission

#### Suspected pneumonia

Of the 1049 infants seen at 6 months of age, 407 (38.8 %) received outpatient care for suspected pneumonia and 92 (8.8 %) required inpatient admission. Maximum duration of inpatient care for suspected pneumonia was 22 days with a median of 7 days.

#### Diarrheal illness

Twenty percent of infants were seen as outpatients for diarrheal illness and 4 % of infants required inpatient admission. Duration of inpatient admission ranged from 1 to 15 days (median 5 days). The majority (96 %) of infants with diarrhea received either medicine or an oral rehydration solution during their illness.

### Factors associated with inpatient admission

#### Suspected pneumonia

Table 2 presents maternal and early infant factors associated with inpatient admission for suspected pneumonia during the first 6 months of life. Exclusive breast feeding at 6 weeks of age (OR 0.39, 95 % CI 0.20 to 0.75) reduced the odds of inpatient admission. Male infants (OR 2.35, 95 % CI 1.40 to 3.96) were twice as likely to require inpatient admission.

**Table 2:** Maternal and early infant predictors of inpatient admission for suspected pneumonia during the first 6 months of life, Ha Nam province, Vietnam (*n* = 1049)

	Univariable regression			Multivariable regression^a^		
Maternal Factors	Odds ratio	95 % CI	*P* value	Odds ratio	95 % CI	*P* value
Demographic factors						
Maternal age (years)	0.96	0.91 to 1.01	0.06	0.95	0.89 to 1.00	0.06
Education						
Primary	Reference	-	-	Reference	-	-
Secondary	0.97	0.53 to 1.78	0.92	0.95	0.48 to 1.91	089
University	1.13	0.54 to 2.35	0.74	1.36	0.56 to 3.33	0.50
Gravidity						
Primgravida	Reference	-	-	Reference	-	-
Multigravida	1.04	0.66 to 1.66	0.86	1.50	0.87 to 2.58	0.15
Main energy source						
Gas	Reference	-	-	Reference	-	-
Firewood/Straw/Hay	0.85	0.45 to 1.61	0.62	0.88	0.45 to 1.72	0.70
Other	0.72	0.22 to 2.39	0.60	0.82	0.18 to 3.68	0.79
Type of toilet						
Flush toilet/septic tank	Reference	-	-	Reference	-	-
Compartment latrine	0.75	0.38 to 1.45	0.39	0.74	0.39 to 1.40	0.36
Pit latrine/none	1.11	0.62 to 1.99	0.72	1.01	0.54 to 1.89	0.98
Main water source						
Rain water	Reference	-	-	Reference	-	-
Well water/river water	2.22	1.04 to 4.73	0.04	2.67	1.05 to 6.77	0.04
Tap/piped water	0.92	0.39 to 2.19	0.85	0.69	0.26 to 1.83	0.46
Nutritional and health status						
Body mass index at enrolment (kg/m^2^)	0.96	0.86 to 1.07	0.44			
Depression early pregnancy	0.74	0.41 to 1.33	0.32			
Depression late pregnancy	1.13	0.58 to 2.20	0.72			
Antenatal practices						
Change of diet when pregnant	1.39	0.81 to 2.38	0.23			
Meat intake during pregnancy (number of times per week)	1.08	0.96 to 1.21	0.23			
Supplements taken during trial intervention						
Daily IFA supplements	Reference	-	-			
Twice weekly IFA supplements	1.28	0.76 to 2.16	0.36	1.10	0.60 to 2.14	0.77
MMN supplements	1.18	0.69 to 2.03	0.55	1.14	0.45 to 1.71	0.69
Micronutrient status in late pregnancy						
Haemoglobin (g/dL)	1.02	0.85 to 1.23	0.80			
Ferritin (log_2_ ug/L)^b^	0.75	0.53 to 1.08	0.12			
B12 (log_2_ pmol/L)^b^	1.22	0.62 to 2.40	0.57			
Folate (log_2_ nmol/L)^b^	0.73	0.41 to 1.29	0.28			
Iodine (log_2_ ug/L)^b^	1.08	0.82 to 1.43	0.60			
Infant factors						
Birthweight (per 100 grams)	0.98	0.98 to 1.00	0.48			
Gestational age at delivery (weeks)	0.88	0.80 to 0.98	0.02	0.92	0.82 to 1.04	0.18
Male sex	2.14	1.35 to 3.39	0.01	2.35	1.40 to 3.96	0.01
Child care practices						
Exclusive breast feeding at six weeks of age	0.44	0.25 to 0.75	0.01	0.39	0.20 to 0.75	0.01
Use of dietary supplements for child in the first six months	0.74	0.40 to 1.38	0.34			

^a^Adjusted for maternal age, education, gravidity, trial intervention, infant sex, gestational age at delivery, type of toilet, water source, main energy source and other factors with statistically significant univariable associations with the outcome

^b^Log_2_ transformed—odds ratio represents a twofold change in ferritin, B12, folate or iodine

#### Diarrheal illness

Table 3 presents maternal and early infant factors associated with inpatient admission for diarrheal illness during the first 6 months of life. Exclusive breast feeding at 6 weeks of age reduced the odds of inpatient admission for diarrheal illness by more than 60 % (OR 0.37, 95 % CI 0.15 to 0.88).

**Table 3:** Maternal and early infant predictors of inpatient admission for diarrheal illness during the first 6 months of life, Ha Nam province, Vietnam (*n* = 1049)

	Univariable regression			Multivariable regression^a^		
Maternal Factors	Odds ratio	95 % CI	*P* value	Odds ratio	95 % CI	*P* value
Demographic factors						
Maternal age (years)	0.94	0.88 to 1.01	0.09	0.92	0.83 to 1.01	0.09
Education						
Primary	Reference	-	-	Reference	-	-
Secondary	1.22	0.50 to 2.97	0.52	1.08	0.40 to 2.88	0.88
University	0.72	0.21 to 2.39	0.59	0.87	0.22 to 3.39	0.84
Gravidity						
Primgravida	Reference	-	-	Reference	-	-
Multigravida	0.76	0.40 to 1.43	0.39	1.22	0.52 to 2.85	0.65
Main energy source						
Gas	Reference	-	-	Reference	-	-
Firewood/Straw/Hay	1.44	0.67 to 3.08	0.35	1.30	0.55 to 3.08	0.55
Other	0.53	0.07 to 3.97	0.54	0.59	0.07 to 4.74	0.50
Type of toilet						
Flush toilet/septic tank	Reference	-	-	Reference	-	-
Compartment latrine	1.20	0.54 to 2.69	0.67	1.16	0.49 to 2.74	0.74
Pit latrine/none	1.04	0.44 to 2.42	0.93	0.82	0.36 to 1.85	0.64
Main water source						
Rain water	Reference	-	-	Reference	-	-
Well water/river water	1.81	0.62 to 5.27	0.28	1.43	0.50 to 4.11	0.50
Tap/piped water	0.59	0.14 to 2.50	0.47	0.57	0.12 to 2.59	0.46
Nutritional and health status						
Body mass index at enrolment (kg/m^2^)	0.89	0.75 to 1.05	0.16			
Depression early pregnancy	0.96	0.44 to 2.11	0.93			
Depression late pregnancy	0.85	0.30 to 2.44	0.77			
Antenatal practices						
Change of diet when pregnant	0.75	0.39 to 1.46	0.40			
Meat intake during pregnancy (number of times per week)	0.98	0.86 to 1.13	0.81			
Supplements taken during trial intervention						
Daily IFA supplements	Reference	-	-	Reference	-	-
Twice weekly IFA supplements	1.44	0.67 to 3.08	0.35	0.72	0.34 to 1.52	0.39
MMN supplements	0.53	0.07 to 3.97	0.54	0.93	0.40 to 2.15	0.86
Micronutrient status in late pregnancy						
Haemoglobin (g/dL)	1.12	0.86 to 1.46	0.41			
Ferritin (log_2_ ug/L)^b^	0.76	0.46 to 1.25	0.28			
B12 (log_2_ pmol/L)^b^	1.05	0.40 to 2.79	0.92			
Folate (log_2_ nmol/L)^b^	1.06	0.45 to 2.47	0.90			
Iodine (log_2_ ug/L)^b^	0.90	0.61 to 1.32	0.59			
Infant Factors						
Birthweight (per 100 grams)	1.03	0.98 to 1.03	0.44			
Gestational age at delivery (weeks)	1.02	0.87 to 1.20	0.78	1.05	0.93 to 1.19	0.46
Male sex	1.51	0.80 to 2.83	0.20	1.68	0.87 to 3.25	0.12
Child care practices						
Exclusive breast feeding at six weeks of age	0.46	0.21 to 1.04	0.05	0.37	0.15 to 0.88	0.03
Use of dietary supplements for child in the first six months	1.23	0.43 to 3.52	0.69			

^a^Adjusted for maternal age, education, gravidity, trial intervention, infant sex, gestational age at delivery, type of toilet, water source, main energy source and other factors with statistically significant univariable associations with the outcome

^b^Log_2_ transformed—odds ratio represents a twofold change in ferritin, B12, folate or iodine

## Discussion

To our knowledge, this is the largest study set in rural Vietnam to present a comprehensive overview of factors associated with inpatient admission for suspected pneumonia and diarrheal illness in early life. Our study demonstrates that the way an infant is fed has a significant effect on the development of severe morbidity in infancy. We demonstrated that exclusive breast feeding in early infancy significantly reduces the risk of inpatient admission for suspected pneumonia or diarrheal illness during the first 6 months of life.

Our findings are consistent with those of previous studies demonstrating the importance of early infant feeding strategies. In a cohort study in the Philippines, Hengstermann et al. demonstrated that exclusively formula fed infants were more likely to be hospitalised for pneumonia and diarrhea, compared to exclusively breast fed infants [12]. Cesar et al. found that infants who were not breast fed were 17 times more likely to be admitted to hospital for pneumonia, compared to breast fed infants [17]. Hipgrave et al. identified increased rates of diarrhoea among children in households receiving infant formula compared with those who did not, [18] and Creek et al. identified that the majority of children hospitalized during a diarrheal outbreak were formula fed [19].

Other studies in developing countries have shown that infants who are formula fed are more likely to be hospitalised with diarrhoeal illness than infants who are exclusively breastfed, and that infants who are not breastfed are up to eight times more likely to die from diarrhoeal disease than breastfed infants [20–26].

Several mechanisms may be responsible for an infant becoming more vulnerable to severe infectious morbidity following the use of formula in early life. Formula may directly expose an infant to pathogens causing diarrhea either through contamination of formula, use of non-clean water sources during the preparation and cleaning of bottles, or inappropriate storage of formula [27–29]. In addition, formula also reduces the growth of beneficial bacteria such as Lactobacillus and Bifidobacteri, leading to a higher pH in the intestine and increased growth of pathogenic bacteria, making the intestinal environment of the child more susceptible to infection [30–32], as well as increasing intestinal permeability and bacterial translocation [33]. Our findings highlight the important immunological and protective benefits that breast feeding provides, in addition to the psychological, social, economic, and environmental benefits [34].

Prevalence of exclusive breast feeding was low in this population, with only one third of infants exclusively breast fed at 6 weeks of age and less than 20 % having ever been exclusively breast fed during the first 6 months of life. Thu et al. have previously reported exclusive breast feeding in rural Vietnam at 3 months of age as 58 % in boys and 65 % in girls [35]. However a recent nutrition surveillance report in Vietnam showed that only 25 % of infants were exclusively breast fed during the first 6 months of life [36]. Reasons for the low prevalence of exclusive breast feeding in Vietnam may include early return to work by mothers with grandparents caring for the infant, insufficient awareness of the benefits of breast feeding over formula feeding, lack of confidence and knowledge of mothers in breast feeding practices, maternal breast feeding misconceptions, family and media influences, social norms around breast feeding practices, and lack of support for women to overcome breast feeding problems [37–39].

The strengths of this study are the large sample size and randomisation of the mothers recruited for the original trial, as well as the comprehensive collection of antenatal and infant variables. Standardised definitions for diarrheal or suspected pneumonia were used to classify cases [15]. Limitations of this study are that the logistics of a large community-based study prevented the prospective collection of clinical and laboratory data at the time of each illness, and therefore factors associated with severe inpatient admission may have been underestimated. Despite these limitations, this is the largest study in rural Vietnam to provide a comprehensive overview of maternal and early life factors associated with severe morbidity in early life. This information will be useful for the development of targeted interventions for the prevention of severe infant morbidity in rural Vietnam.

## Conclusion

Inpatient admission for suspected pneumonia or diarrheal illness in early infancy is reduced with exclusive breast feeding. There is an urgent need to develop strategies to address barriers to exclusive breast feeding in rural Vietnam, such as increased exclusive breast feeding promotional and educational activities at the primary health care level; improved support for mothers to exclusively breast feed from birth up until 6 months of age; eradication of common breast feeding myths; and strengthening of policies around promotion of exclusive breast feeding.

## Abbreviations

CI: Confidence interval
CHS: Commune health station
(DSM IV): Diagnostic and statistical manual of mental disorders, 4th edition
Hb: Haemoglobin
OR: Odds ratio
SD: Standard deviation
UNIMMAP: UNICEF/WHO/UNU international multiple micronutrient preparation
WHO: World health organisation
